# Fasting plasma glucose variability and all-cause mortality among type 2 diabetes patients: a dynamic cohort study in Shanghai, China

**DOI:** 10.1038/srep39633

**Published:** 2016-12-22

**Authors:** Dongli Xu, Hong Fang, Wanghong Xu, Yujie Yan, Yinan Liu, Baodong Yao

**Affiliations:** 1 Shanghai Minhang Center for Disease Control and Prevention, Shanghai, China; 2 Department of Epidemiology, School of Public Health, Fudan University, and The Key Laboratory of Public Health Safety of Ministry of Education (Fudan University), Shanghai, China

## Abstract

The study aims to examine whether the variation of fasting plasma glucose (FPG), represented by coefficient of variation (CV), independently predicts all-cause mortality among Chinese type 2 diabetes patients. This retrospective cohort study was designed based on a standardized electronic management system of diabetes patients in Shanghai, China. 8871 type 2 diabetes patients were enrolled between 1 January 2007 and 31 December 2007 and were followed-up for all-cause mortality until 31 December 2014. All patients were grouped by the quartiles of CV of FPG. 1136 patients deceased during following-up. After adjusting for other risk factors, CV of FPG was not independently associated with all-cause mortality. Stratified analysis by mean FPG levels (<7 mmol/L and ≥7 mmol/L) observed a significant modifying effect of CV of FPG (*P* for interact test <0.01). CV of FPG was independently associated with all-cause mortality in patients whose glucose control was poor, with the HRs (95% CI) for the second, third, fourth vs first quartiles of CV of FPG being 1.23(0.94–1.61), 1.23(0.94–1.61), and 1.63(1.25–2.13), respectively. Our results suggest that variability of FPG may be an important predictor of mortality among type 2 diabetes in China, particularly for those with their glycemic status uncontrolled.

The global burden of diabetes mellitus has been rising dramatically over the past two decades. It is estimated that around the world more than 552 million people will have type 2 diabetes by 2030^1^. The presence of type 2 diabetes increases the risk of death^2^,^3^,^4^. Reducing diabetes-related premature death across populations requires better management and control of diabetes and other cardiovascular risk factors.

A number of studies have examined the relationship of mortality in type 2 diabetes patients with some risk factors such as estimated glomerular filtration rate (GFR), glycated hemoglobin A1C, and LDL cholesterol^5^,^6^. However, very few studies have examined the predictive value of glycemic variability. In recent years, several studies have raised concerns on the possible adverse effects of glycemic variability in diabetes patients^7^,^8^,^9^. Data from the Verona Diabetes Study and the Taichung Diabetes Study have showed that glycemic variability was an independent predictor of mortality in type 2 diabetes patients^10^,^11^,^12^. However, these previous studies have not examined the possible confounding and modifying effect of glycemic status of control in associations between variability of FPG and mortality. Moreover, no evidence is available on the association of glycemic variability with mortality in Chinese diabetes patients in Mainland of China.

In this study, we took advantage of the subjects from the standardized electronic management system in Minhang district of Shanghai, China, as a dynamic cohort to investigate the association of glycemic variability with all-cause mortality among Chinese patients with type 2 diabetes.

## Results

By the end of follow-up, a total of 1136 type 2 diabetes patients (574 men, 562 women) were confirmed dead, with overall mortality rate being 19.91/1,000 person-years (23.09/1,000 in men and 17.46/1,000 in women). Cardiovascular disease was the leading cause of death (n = 425), followed by cancer (n = 309) and diabetes (n = 226). Table 1 shows the comparisons of baseline socio-demographic and clinical factors of survivors and the deceased after an average of 6.43 years of following-up. Compared with the survival patients, the deceased patients were more likely to be male and older, had a longer duration of diabetes, lower mean of BMI, higher mean of SBP, higher mean FPG and CV of FPG, and more frequently used insulin.

Table 2 presents baseline socio-demographic and clinical factors in subgroups of patients by quartiles of CV of FPG. Along with the increasing quartiles of CV of FPG, decreasing average ages and significantly increasing baseline FPG levels and duration of diabetes were observed in our participants.

**Figure 1:**
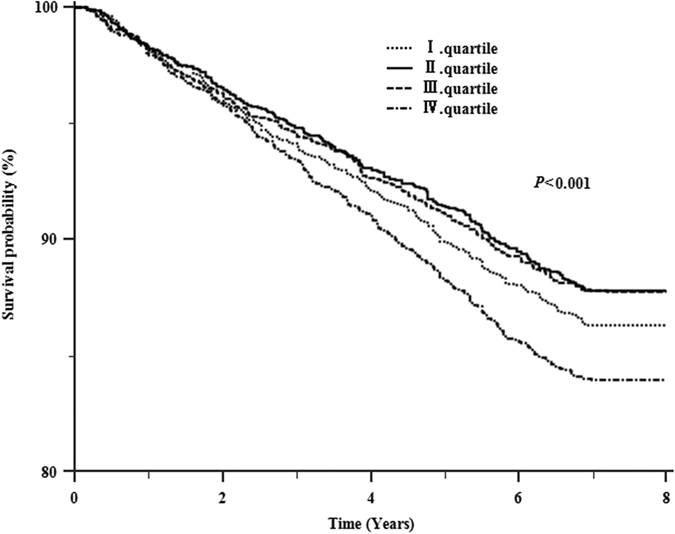
Kaplan-Meier estimates of survival probability in 8871 type 2 diabetes patients from 1 January 2007 through 31 December 2014. Patients were grouped by quartiles of the CV of FPG within the first one year of following-up. The log-rank tests revealed significant differences in survival among quartiles of the CV of FPG (*P* < 0.001).

As shown in Fig. 1, patients in the top quartile of CV of FPG experienced higher mortality than patients of the other quartiles(*P *< 0.001) during the 8-year following-up period. No significant difference was observed in survival among other quartiles groups during the following-up.

Table 3 shows HRs for death from all causes in subjects grouped by quartiles of CV of FPG. Compared to patients with the lowest quartile, age-gender adjusted HRs (95%CI) in the second, third and highest CV of FPG quartiles were 0.89 (0.75–1.05), 0.95 (0.81–1.13) and 1.24 (1.06–1.45), respectively. When further adjusting for duration of diabetes, methods of DM treatment, baseline smoking status, physical activity, SBP, DBP, family history and BMI categories (model 2), the effect of CV of FPG attenuated, but still remained statistically significant (HR 1.18, 95%CI 1.01–1.39). Further adjusting for baseline FPG showed that it was baseline FPG, but not CV of FPG, was an independent predictor of mortality. Other predictors of mortality included male sex (all three models), age (all three models), BMI < 18.5 (model 2 and model 3), BMI ≥ 30.0 (model 2 and model 3) and insulin treatment (model 2 and model 3). Sensitivity analysis by excluding participants who died during the first 2 years of follow-up did not find that the results changed substantially. We also excluded patients who died due to external (injury-related) causes (n = 42). There was little difference seen for the effect of fasting plasma glucose variability on all-cause mortality. Then we investigated associations between fasting plasma glucose variability and cause-specific mortality (Supplementary Table 1).

We found significant interaction effects of FPG-CV and mean FPG (*P* for interact test <0.01). In order to rule out the effect of glucose status of control on variability of FPG, we performed cox regression models stratified by mean FPG levels of subjects (<7 mmol/L vs. ≥7 mmol/L). As shown in Table 4, in the group with mean FPG < 7 mmol/L, CV of FPG was not associated with the risk of death, while in the group with FPG ≥ 7 mmol/L, CV of FPG was an independent predictor of mortality in all three models, with HRs (95%CI) for all-cause mortality in model 3 being 1.23(0.94–1.61), 1.23(0.94–1.61) and 1.63(1.25–2.13), respectively, across increasing quartile groups comparing with the lowest quartile group.

## Discussion

In this first large-scale study to investigate the association of glycemic variability with all-cause mortality among type 2 diabetes patients in Mainland China, we found that variability of FPG was independently associated with all-cause mortality in type 2 diabetes patients whose glucose level was poorly controlled, but not a predictor of all-cause mortality in patients with their glucose well controlled.

In previous studies^10^,^13^,^14^, the CV of FPG was observed a significant predictor for all-cause mortality in type 2 diabetes patients. The Taichung Diabetes Study^10^ measured glycemic variability by computing annual CV of all FPG measurements within each year, and showed that annual CV of FPG was independently associated with all-cause mortality in patients with type 2 diabetes aged 30 years and over. The Verona Diabetes Study^13^ found that FPG variability, as assessed by CV of FPG over a period of three years, was an independent predictor of all-cause in patients with type 2 diabetes aged 56–74 years. In our analysis, we examined the relationship between variability of FPG and all-cause mortality in subgroups stratified by glucose status of control. The present results showed that variability of FPG was only independently associated with a higher risk of subsequent all-cause death in those with their glycemic status uncontrolled. A large national survey in China showed that the mean BMI was 25.2 for men with previously diagnosed diabetes and the mean BMI was 24.6 for women with previously diagnosed diabetes^15^. The mean BMI was 23.9 for type 2 diabetes patients in our study, which was lower than that of previously diagnosed diabetes patients in the national survey^15^. However, the duration of diabetes was not indicated in the national survey, and the mean duration of diabetes in our study was 13.0 years.

Several potential mechanisms may explainthe association between glycemic variability and all-cause mortality. First, glycemic variability might be an indicator of irregular compliance to therapy due to a variety of reasons (poor healtheducation, insufficient awareness of the severity of the disease)^13^. Our study had considered the effect of adherence to therapy or diet guideline on all-cause mortality. The proportion of adherence to therapy or diet guideline for different groups of CV of FPG was similar. And adherence to therapy or diet guideline wasn’t in the final regression model. Second, glycemic variability might be an indication of poor health, comorbidity, or complication that results in the increase of mortality. A previous study^10^ had considered the associations of baseline comorbidity or complication with mortality and they can explain only a small amount of these associations. Third, glucose fluctuation has been shown to cause over production of superoxide that is a key risk factor in the pathogenesis of diabetes complications^16^,^17^. The increases in diabetes complications further result in the increase of mortality.

Our study has some strengths, including retrospective cohort study design, a large number of diabetes patients, long-term of following-up and standardized procedure for data collection according to the Diabetes Prevention Guide. In addition, to our best knowledge, it is the first study to examine the relationship between variability of FPG and all-cause mortality in subgroups stratified by glycemic status of control. However, this study has several limitations. First, unlike RCTs, FPG measurements in this study were derived from clinical follow-ups, thus the frequency of FPG measurements and the intervals between measurements varied across patients. Although we adjusted for the effect of the frequency of FPG measurements on variability, the difference of intervals between FPG measurements had not been fully addressed. Second, we didn’t adjust for baseline comorbidity and presence of diabetic complications due to lacking of the information, which may have biased our results. Finally, baseline glycated hemoglobin determinations were not available for all participants and they have been observed as one of the independent risk factors of macrovascular events which may result in the increase of mortality^18^.

In conclusion, glycemic variability was a potent independent predictor of all-cause mortality in type 2 diabetes patients whose glucose was poorly controlled. Tight control of FPG variability may provide further protection against subsequent death in type 2 diabetes. Further randomized, controlled trials investigating the favorable effectsof an intervention of maintaining the stable glycemia are needed to confirm our results and elucidate the direct causality.

## Methods

### Study population and data source

This retrospective cohort study was a population-based cohort study of diabetes patients enrolled in a standardized management system of diabetes in Shanghai, China^19^. The standardized management system for diabetes outpatients, as a basic community health service, was carriedout since 2004 in Minhang district, one of 17 administrative divisions of Shanghai, China. According to the Chinese National Diabetes Prevention Guide, the standardized management was designed as a system to carry out regular following-up of patients by General Practitioners. All diabetes patients were diagnosed based on the 1999 criteria of the World Health Organization (WHO)^20^, and were followed up regularly once per month or every 3 months, depending on the glycemic status of control. At the following-up visits, the status of adherence to therapy or diet guideline had been evaluated by General Practitioners. All following-up data was recorded in electronic health records (eHR) database^21^.

### Study design

**Figure 2:**
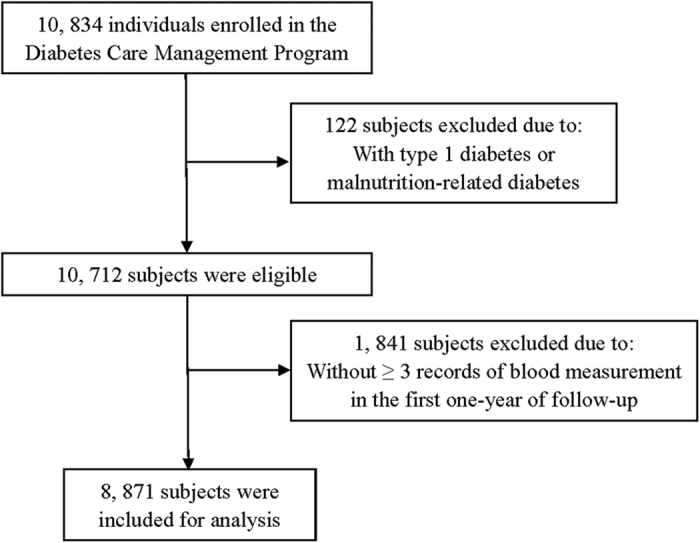
Flow chart of recruitment procedures of study participants for the current study.

We included type 2 diabetes patients enrolled in the registry between 1 January 2007 and 31 December 2007. 122 subjects with type 1 diabetes or malnutrition-related diabetes were excluded, and 10,712 (4763 men, 5949 women) type 2 diabetes patients were eligible (Fig. 2). And 1841 subjects without ≥3 records fasting plasma glucose (FPG) in the first one-year of following-up to analyze variability were excluded. Then 8871 (3909 men, 4962 women) subjects were finally included in the analysis. Written informed consent was obtained for all study participants when they were enrolled in the registry. The study was approved by the Institutional Review Board of Minhang Center for Disease Control and Prevention. The methods were carried out in accordance with the approved guidelines.

Date of diabetes patients registered into the standardized managementwas defined as the index date. The survival status of patients up to 31 December 2014 was ascertained according to the recordsin the management system. We also identified the survival status through record-linkage of the eHR system with the Vital Statistics system in Minhang district, which is part of national Disease Surveillance Point System^22^. We used the underlying cause of death ICD-10 code on the Vital Statistics system to group causes of death. The categories were as follows: diabetes (E10–E14), cardiovascular disease (I00–99), cancer (C00–97), external (injury-related) causes (V00–Y89) and all other codes. The end points of follow-up were defined as the time when he/she was dead or censored (due to lost to follow up). 1136 deaths were identified in this cohort.

### Measurements

The demographic characteristics (birth date and sex), measurements of body height, weight, BMI, systolic blood pressure (SBP), diastolic blood pressure (DBP), FPG levels, family history, diagnosis date of diabetes, methods of DM treatment (oral hypoglycemic drug, insulin injection, both, or diet or exercise) and adherence to therapy or diet guideline were obtained from the eHR database for all diabetes patients. Duration of diabetes was calculated as the calendar date when he/she was dead or censored minus the calendar date of diagnosis with type 2 diabetes. A number of studies suggested a protective effect of overweight in patients with type 2 diabetes (obesity paradox)^23^,^24^. Adjusting for BMI as a continuous may result in some bias, and we adjusted for BMI on mortality using BMI categories. BMI categories were defined as follows: <18.5, 18.5 to 24.9 (reference), 25.0 to 29.9 and ≥30.0, respectively^15^.

### Statistical analysis

The variability (coefficient of variation, CV) of FPG measurements from diabetes patients visits within the first year of follow-up for each patientwas calculated. Considering that the frequency of visits may affect the evaluation of variability, the CV of FPG was adjusted by dividing by the square root of the ratio of total visits divided by total visits minus 1^25^,^26^. All patients were classified into four groups by quartiles of CV of FPG.

Unpaired Student’s t tests, χ^2^ tests, or one-way analysis of variance were used to make comparisons between/among subgroups. Univariate survival analysis was performed by Kaplan-Meier method and log-rank test. Multivariable survival analysis was conducted using a Cox regression model by considering all-cause death as anevent. Hazard ratios (HRs) and 95% confidence intervals (CI) were calculated based on:(1) model 1: adjusted for age and gender; (2) model 2: adjusted for variables in model 1 plus duration of diabetes, baseline smoking status, physical activity, family history, blood pressure, BMI categories and methods of diabetes treatment; and (3) model 3: adjusted for variables in model 2 plus baseline FPG. Interaction of FPG-CV and mean FPG was probed by adding their product terms into the full model using the likelihood ratio test for significance.

All analyses were performed with SAS version 9.1 (SAS, Cary, NC); all *P* valueswere 2-tailed, and *P* value < 05 was considered statistically significant.

## Additional Information

**How to cite this article**: Xu, D. *et al*. Fasting plasma glucose variability and all-cause mortality among type 2 diabetes patients: a dynamic cohort study in Shanghai, China. *Sci. Rep.* **6**, 39633; doi: 10.1038/srep39633 (2016).

**Publisher's note:** Springer Nature remains neutral with regard to jurisdictional claims in published maps and institutional affiliations.

## Supplementary Material

Supplementary Table 1

## Figures and Tables

**Table 1:** The comparisons of baseline socio-demographic and clinical factors of survivors and the deceased included in the analysis.

Variables	All patients (n = 8871)	Mortality Status		*P-*values
		Deceased (n = 1136)	Survivors (n = 7735)	
**Sex**				
Male, n (%)	3909 (44.06)	574 (50.53)	3335 (43.12)	<0.001
Age (years)	71.93 ± 11.02	81.04 ± 9.37	70.59 ± 10.60	<0.001
BMI, kg/m^2^	23.92 ± 3.52	23.45 ± 3.74	23.99 ± 3.49	<0.001
Baseline SBP, mmHg	130.79 ± 10.79	131.85 ± 11.13	130.64 ± 10.73	<0.001
Baseline DBP, mmHg	80.42 ± 6.54	80.01 ± 6.62	80.47 ± 6.52	<0.05
**Lifestyle behaviors**				
Smoking, n (%)	1517 (17.10)	230 (20.25)	1287 (16.64)	<0.01
Physical activity, n (%)	3059 (34.48)	362 (31.87)	2697 (34.86)	<0.05
**Diabetes-related variables**				
Duration of diabetes (years)	13.02 ± 5.71	14.43 ± 6.69	12.81 ± 5.52	<0.001
Methods of DM treatment				<0.001
No medication, n (%)	1499 (16.90)	152 (13.38)	1347 (17.41)	
One oral hypoglycemic medicine, n (%)	4957 (55.88)	636 (55.99)	4321 (55.86)	
Two oral hypoglycemic medicines, n (%)	1622 (18.28)	198 (17.43)	1424 (18.41)	
≥3 oral hypoglycemic medicines, n (%)	67 (0.76)	7 (0.62)	60 (0.78)	
Insulin only, n (%)	537 (6.05)	109 (9.60)	428 (5.53)	
Insulin + oral hypoglycemic medicines, n (%)	189 (2.13)	34 (2.99)	155 (2.00)	
Mean FPG (mmol/L) (within the first year)	7.12 ± 1.29	7.27 ± 1.53	7.09 ± 1.25	<0.001
Baseline FPG level	7.41 ± 2.10	7.50 ± 2.38	7.39 ± 2.06	0.099

Note: Abbreviations: BMI, body mass index; SBP, systolic blood pressure; DBP, diastolic blood pressure; FPG, fasting plasma glucose; CV, coefficientof variation.

**Table 2:** Baseline factors grouped by quartiles of the coefficient of variation of FPG levels.

Variables (% or mean ± SD)	Baseline quartiles of FPG-CV				*P-*value
	1 (lowest)	2	3	4 (highest)	
Quartiles range	≤4.25	4.25~7.75	7.75~13.45	>13.45	
n	2215	2220	2218	2218	
Sex					0.004
Male, n (%)	1025 (46.28)	993 (44.73)	910 (41.03)	981 (44.23)	
Female, n (%)	1190 (53.72)	1227 (55.27)	1308 (58.97)	1237 (55.77)	
Age (years)	72.27 ± 10.90	72.31 ± 10.96	71.70 ± 10.84	71.45 ± 11.36	0.019
BMI, kg/m^2^	23.99 ± 3.15	23.95 ± 3.32	23.94 ± 3.28	23.81 ± 4.24	0.348
Baseline SBP, mmHg	130.91 ± 10.52	130.61 ± 10.45	130.65 ± 10.98	130.99 ± 11.19	0.578
Baseline DBP, mmHg	80.41 ± 6.41	80.40 ± 6.31	80.40 ± 6.61	80.46 ± 6.81	0.986
Lifestyle behaviors					
Smoking, n (%)	370 (16.70)	378 (17.04)	366 (16.50)	403 (18.16)	0.457
Physical activity, n (%)	784 (35.38)	759 (34.20)	768 (34.63)	748 (33.72)	0.686
**Diabetes-related variables**					
Duration of diabetes (years)	12.83 ± 5.55	12.70 ± 5.30	12.85 ± 5.50	13.70 ± 6.39	<0.001
Methods of DM treatment					<0.001
No medication, n (%)	466 (21.04)	438 (19.73)	358 (16.14)	237 (10.69)	
One oral hypoglycemic medicine, n (%)	1261 (56.93)	1296 (58.38)	1242 (56.00)	1158 (52.21)	
Two oral hypoglycemic medicines, n (%)	339 (15.30)	353 (15.90)	438 (19.75)	492 (22.18)	
≥3 oral hypoglycemic medicines. n (%)	15 (0.68)	11 (0.50)	22 (0.99)	19 (0.86)	
Insulin only, n (%)	100 (4.51)	96 (4.32)	120 (5.41)	221 (9.96)	
Insulin + oral hypoglycemic medicines, n (%)	34 (1.53)	26 (1.17)	38 (1.71)	91 (4.10)	
Baseline FPG level	6.66 ± 0.80	6.76 ± 0.94	7.23 ± 1.46	8.97 ± 3.25	<0.001
Mean of FPG (mmol/L) (within the first year)	6.63 ± 0.76	6.70 ± 0.80	7.07 ± 1.16	8.05 ± 1.68	<0.001

Note: Abbreviations: FPG, fasting plasma glucose; CV, coefficientof variation; BMI, body mass index; SBP, systolic blood pressure; DBP, diastolic blood pressure.

**Table 3:** The hazard ratios (HRs) of all-cause mortality grouped by quartiles of the coefficient of variation of FPG in type 2 diabetes patients enrolled in diabetes care management program.

	Baseline quartiles of CV of FPG			
	1 (lowest)	2	3	4 (highest)
n	2215	2220	2218	2218
Deaths from all-causes, n (%)	285 (12.87)	254 (11.44)	257 (11.59)	340 (15.33)
Model 1	1.00	0.89 (0.75–1.05)	0.95 (0.81–1.13)	1.24 (1.06–1.45)**
Model 2	1.00	0.87 (0.73–1.03)	0.94 (0.79–1.11)	1.18 (1.01–1.39)*
Model 3	1.00	0.86 (0.73–1.02)	0.92 (0.78–1.09)	1.10 (0.93–1.31)

Note: Abbreviations: FPG fasting plasma glucose, CV coefficient of variation. Model 1: adjusted for age and gender. Model 2: adjusted for age, gender, duration of diabetes, smoking, physical activity, methods of DM treatment, SBP, DBP, family history and BMI categories. Model 3: adjusted for variables in the model 2 plus baseline FPG. ^*^P < 0.05; **P < 0.001.

**Table 4:** The hazard ratios (HRs) of all-cause mortality grouped by quartiles of the coefficient of variation of FPG in type 2 diabetes patients stratified by mean FPG.

	Mean FPG < 7 (n = 5249)				Mean FPG ≥ 7 (n = 3622)			
	1 (lowest)	2	3	4 (highest)	1 (lowest)	2	3	4 (highest)
Quartiles range of CV of FPG	≤3.33	3.33~5.84	5.84~9.49	>9.49	≤7.29	7.29~12.31	12.31~19.12	>19.12
n	1318	1306	1312	1313	906	905	906	905
Deaths from all-causes, n (%)	168 (12.75)	167 (12.79)	143 (10.90)	167 (12.72)	100 (11.04)	112 (12.38)	121 (13.36)	158 (17.46)
Model 1	1.00	1.01 (0.81–1.25)	0.82 (0.66–1.03)	0.98 (0.79–1.21)	1.00	1.25 (0.95–1.63)	1.28 (0.98–1.67)	1.75 (1.36–2.25)**
Model 2	1.00	1.00 (0.80–1.23)	0.82 (0.65–1.02)	0.96 (0.78–1.19)	1.00	1.23 (0.94–1.61)	1.25 (0.96–1.62)	1.67 (1.30–2.15)**
Model 3	1.00	0.99 (0.80–1.23)	0.82 (0.65–1.02)	0.95 (0.77–1.18)	1.00	1.23 (0.94–1.61)	1.23 (0.94–1.61)	1.63 (1.25–2.13)**

Note: Abbreviations: FPG fasting plasma glucose, CV coefficientof variation. Model 1: adjusted for age and gender. Model 2: adjusted for age, gender, duration of diabetes, smoking, physical activity, methods of DM treatment, SBP, DBP, family history and BMI categories. Model 3: adjusted for variables in the model 2 plus baseline FPG.^*^P < 0.01; ^**^P < 0.001.
