# Supplementary material for: Fasting plasma glucose variability and all-cause mortality among type 2 diabetes patients: a dynamic cohort study in Shanghai, China
Source: Sci Rep. 2016 Dec 22;6:39633. doi: 10.1038/srep39633 (PMC5177938; doi:10.1038/srep39633)
Supplement: Supplementary Table 1 [file srep39633-s1.pdf]

Fasting plasma glucose variability and all-cause mortality among type 2 diabetes patients: a dynamic cohort study in Shanghai, China

Dongli Xu[1], Hong Fang[1], Wanghong Xu[2], Yujie Yan[1], Yinan Liu[1]& Baodong Yao[1*]

[1]Shanghai Minhang Center for Disease Control and Prevention, Shanghai, China.

[2]Department of Epidemiology, School of Public Health, Fudan University, and The Key Laboratory of Public Health Safety of Ministry of Education (Fudan University), Shanghai, China. [*] Correspondence and requests for materials should be addressed to B.D.Y. (email: 09211020010@fudan.edu.cn)

Variables	**Diabetes-related Mortality HR (95%CI)**	**Cardiovascular disease-related mortality HR (95%CI)**	**Cancer-related mortality HR (95%CI)**
Model 1			
CV of FPG (%)			
≤4.25	1.00	1.00	1.00
4.25~7.75	0.84(0.55-1.28)	0.95(0.71-1.28)	0.96(0.66-1.38)
7.75~13.45	0.85(0.55-1.31)	0.95(0.70-1.29)	1.21(0.85-1.72)
>13.45	1.51(1.03-2.20)*	1.18(0.88-1.57)	1.41(1.00-1.98)
Model 2			
CV of FPG (%)			
≤4.25	1.00	1.00	1.00
4.25~7.75	0.82(0.53-1.25)	0.95(0.71-1.28)	0.90(0.62-1.30)
7.75~13.45	0.84(0.55-1.30)	0.95(0.70-1.29)	1.18(0.83-1.67)
>13.45	1.42(0.97-2.08)	1.18(0.88-1.57)	1.39(0.99-1.96)
Model 3			
CV of FPG (%)			
≤4.25	1.00	1.00	1.00
4.25~7.75	0.81(0.53-1.25)	0.95(0.70-1.27)	0.89(0.62-1.30)
7.75~13.45	0.83(0.54-1.28)	0.93(0.69-1.27)	1.17(0.83-1.67)
>13.45	1.32(0.88-2.00)	1.07(0.78-1.47)	1.38(0.95-2.00)

Supplementary Table 1. The hazard ratios (HRs) of cause-specific mortality grouped by quartiles of the coefficient of variation of FPG in type 2 diabetes patients enrolled in diabetes care management program. Note: Abbreviations: CI, confidence interval; FPG, fasting plasma glucose; CV, coefficient of variation. Model 1: adjusted for age and gender. Model 2: adjusted for age, gender, duration of diabetes, smoking, physical activity, methods of DM treatment, SBP, DBP, family history and BMI categories. Model 3: adjusted for variables in the model 2 plus baseline FPG. * P<0.05.
